# Possible role of the Nipah virus V protein in the regulation of the interferon beta induction by interacting with UBX domain-containing protein1

**DOI:** 10.1038/s41598-018-25815-9

**Published:** 2018-05-16

**Authors:** Shotaro Uchida, Ryo Horie, Hiroki Sato, Chieko Kai, Misako Yoneda

**Affiliations:** Laboratory Animal Research Center and International Research Center for Infectious Diseases, Institute of Medical Science, The University of Tokyo, Tokyo, Japan

## Abstract

Nipah virus (NiV) is a highly pathogenic paramyxovirus that causes lethal encephalitis in humans. We previously reported that the V protein, one of the three accessory proteins encoded by the P gene, is one of the key determinants of the pathogenesis of NiV in a hamster infection model. Satterfield B.A. *et al*. have also revealed that V protein is required for the pathogenicity of henipavirus in a ferret infection model. However, the complete functions of NiV V have not been clarified. In this study, we identified UBX domain-containing protein 1 (UBXN1), a negative regulator of RIG-I-like receptor signaling, as a host protein that interacts with NiV V. NiV V interacted with the UBX domain of UBXN1 via its proximal zinc-finger motif in the C-terminal domain. NiV V increased the level of UBXN1 protein by suppressing its proteolysis. Furthermore, NiV V suppressed RIG-I and MDA5-dependent interferon signaling by stabilizing UBXN1 and increasing the interaction between MAVS and UBXN1 in addition to directly interrupting the activation of MDA5. Our results suggest a novel molecular mechanism by which the induction of interferon is potentially suppressed by NiV V protein via UBXN1.

## Introduction

Nipah virus (NiV) is an emerging zoonotic virus which belongs to the genus *Henipavirus* in the family *Paramyxoviridae*, and was first identified as the pathogen that caused an outbreak of fatal encephalitis in humans in Malaysia and Singapore in 1999^1,2^. Since 2001, outbreaks of NiV have occurred sporadically almost every year in India, Bangladesh, and the Philippines^3–7^, and high mortality rates of around 40–70% in humans have been reported^4,7^. Serological evidence of henipavirus infections among humans and bats has also been reported in regions where no outbreaks have yet occurred^8–11^, implying that other areas are threatened with future outbreaks of henipavirus infections. Therefore, it is important to understand the molecular mechanism underlying the severe pathogenesis of henipavirus infections to allow the development of effective treatments.

The genome of NiV is a negative-strand nonsegmented RNA containing six genes encoding structural proteins: nucleocapsid (N), phosphoprotein (P), matrix protein (M), fusion protein (F), glycoprotein (G), and polymerase (L). The P gene encodes three additional accessory proteins: V, W, and C proteins^2,12^. The RNA of the P gene is edited to generate the V and W genes, when additional guanines are inserted at the RNA editing site due to the stuttering of the RNA polymerase during mRNA transcription^12^. Thus, the P, V, and W proteins share a common N-terminal domain, but each has a unique C-terminal domain. The C protein is encoded in an alternative reading frame of the P gene. We have previously reported that the V and C proteins play key roles in the severe pathogenicity of NiV in a hamster infection model^13^. Satterfield B. A. *et al*. have also reported that V protein is a determinant of the disease course of NiV infection, and C protein contributes to the respiratory disease in a ferret infection model^14,15^. Mathieu C. *et al*. have revealed that C protein contributed to the virulence by regulating the early host proinflammatory response^16^. Recently, Marsh G.A. *et al*. isolated Ceder virus (CedPV) from Australian bats as a novel member of henipaviruses. CedPV lacked the RNA editing site for the V protein expression, and was attenuated in ferrets and guinea pigs which are susceptible to NiV and Hendra virus (HeV)^17^. It also indicates that V protein is a critical for the pathogenesis of henipaviruses.

Previous studies have demonstrated that henipavirus V proteins suppress the host antiviral response by targeting multiple host proteins. Henipavirus V proteins interact with MDA5 to inhibit the activation of the interferon β (*IFNB*) promoter^18,19^. NiV V binds to the phosphatase PP1 to suppress the dephosphorylation of MDA5 and thereby its activation^20^. Henipavirus V proteins also interact with LGP2 to suppress the RIG-I-dependent induction of IFN^21^. NiV V also blocks signaling through Toll-like receptors 7/9 (TLRs 7/9) and inhibitor of κB kinase ε to suppress IFN induction^22,23^. V proteins of NiV and HeV interacts with signal transducer and activator of transcription 1 and 2 (STAT1 and STAT2) in IFN-responsive signaling pathway, through its N-terminal domain, which it shares with the P and W proteins, and prevents their activation and nuclear accumulation^24,25^. During viral replication, NiV V negatively regulates minigenome replication^26^. These reports have revealed that V protein interacts with multiple host proteins to contribute to the severe pathogenicity of henipavirus, and to elucidate the unknown molecular mechanism underlying the pathogenicity of henipavirus, the host molecule interacting with henipavirus V proteins should be further investigated.

In this study, we looked for unknown host proteins that interact with NiV V and identified UBX domain-containing protein 1 (UBXN1). We then analyzed the effects of the interaction between NiV V and UBXN1.

## Results

### Identification of a protein that interacts with NiV V protein

**Figure 1:**
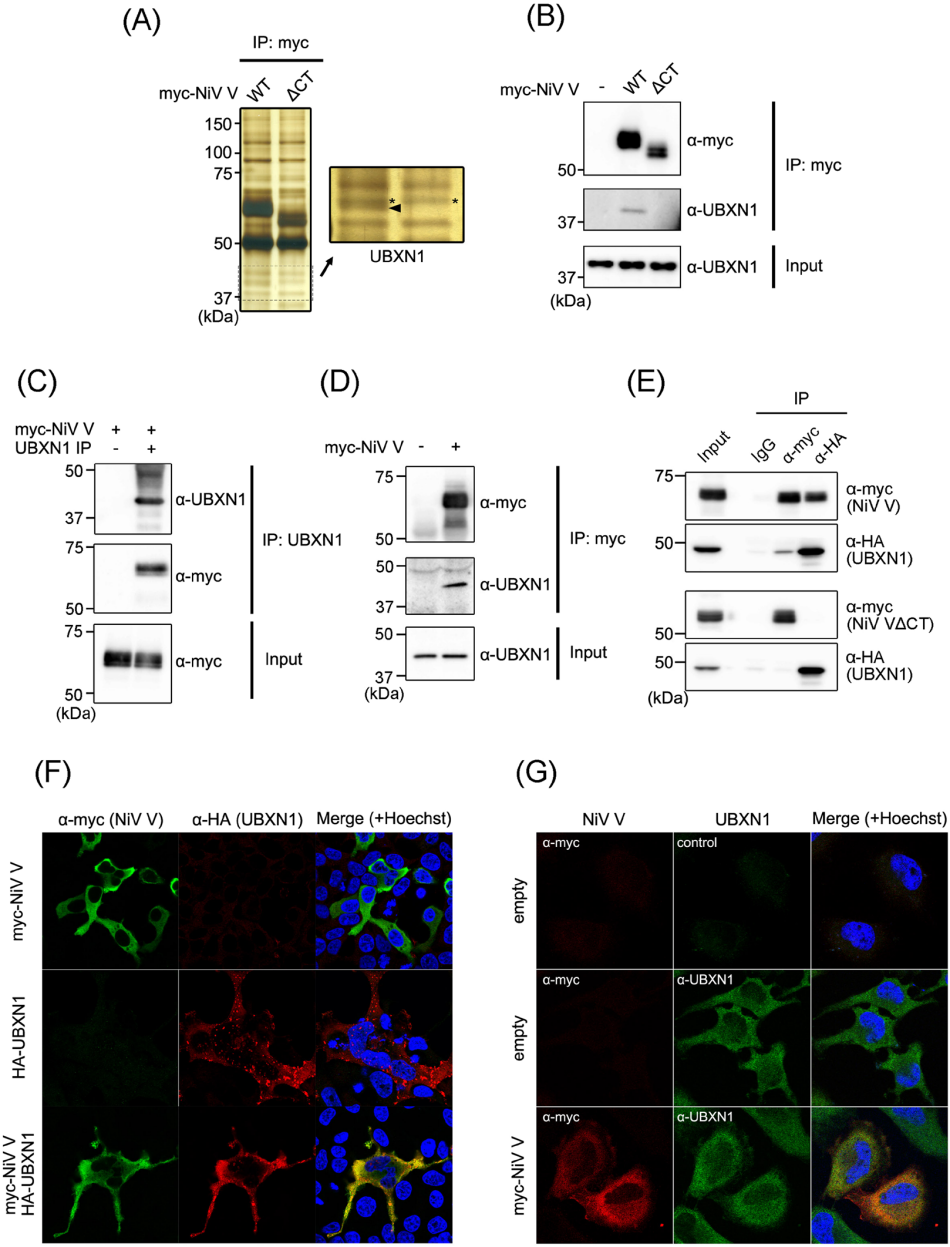
Identification of a protein that interacts with NiV V. (**A**) Myc-tagged NiV V and a mutant lacking the C-terminal domain (ΔCT) were expressed in HEK293T cells. At 48 h posttransfection, an immunoprecipitation assay was performed, and the precipitated proteins were detected with silver staining. The band indicated by the arrowhead was analyzed with mass spectrometry. *Nontargeted bands. (**B**) The immunoprecipitation assay was performed as described in (**A**), and the precipitated proteins were detected with western blotting. (**C**) Myc-tagged NiV V was expressed in HEK293T cells, and at 48 h posttransfection, an immunoprecipitation assay was performed using UBXN1-specific antibody. The precipitated proteins were detected with western blotting. (**D**) Myc-tagged NiV V expressed in HeLa cells was immunoprecipitated, and the precipitated proteins were detected with western blotting. (**E**) Myc-tagged NiV V or ΔCT were expressed together with HA-tagged UBXN1 in HEK293T cells, and after 48 h, an immunoprecipitation assay was performed with anti-myc or anti-HA antibody. The precipitated proteins were detected with western blotting. (**F**) NiV V and HA-tagged UBXN1 were expressed in HEK293T cells, and after 24 h, an indirect immunofluorescence assay was performed. The subcellular localization of NiV V and UBXN1 was observed with confocal microscopy. (**G**) NiV V was expressed in HeLa cells, and the subcellular localization of NiV V and endogenous UBXN1 was examined by an indirect immunofluorescence assay. The gel and blots presented in (**A**–**E**) were cropped from different images to improve clarity. Full-length gel and blots are presented in Supplementary Figure S1.

To identify host proteins that specifically interact with the unique C-terminal domain of NiV V, which is not shared with the P or W protein, myc-tagged wild-type NiV V and a mutant lacking the C-terminal domain (ΔCT) were expressed in HEK293T cells and immunoprecipitated with an anti-myc antibody. Two protein bands coimmunoprecipitated with wild-type NiV V, but not with ΔCT were detected (Supplementary Fig. 1A). One of the proteins with a mass of approximately 42.56 kDa was identified as UBX domain-containing protein 1 (UBXN1) by a mass-spectrometric analysis (Fig. 1A). To confirm the result of the mass-spectrometric analysis, the same immunoprecipitation assay was followed by western blotting with an anti-myc antibody and an anti-UBXN1 antibody. UBXN1 was detected as the protein binding to wild-type NiV V, but not to ΔCT (Fig. 1B). The interaction was also verified by the coimmunoprecipitation of NiV V with the endogenous UBXN1 (Fig. 1C). In HeLa cells, UBXN1 was also coimmunoprecipitated with NiV V, indicating that UBXN1 is a binding partner of NiV V in various cell types (Fig. 1D). To confirm the requirement of the C-terminal zinc-finger domain for the interaction, myc-tagged wild-type NiV V or ΔCT were coexpressed with HA-tagged UBXN1 in HEK293T cells and immunoprecipitated with either anti-myc antibody or anti-HA antibody. The wild-type NiV V, but not ΔCT, was coimmunoprecipitated with UBXN1 (Fig. 1E). To confirm their intracellular interaction, an immunofluorescence assay was also performed after NiV V or UBXN1 was expressed in HEK293T cells. As previously reported, NiV V was mainly localized in cytoplasm (Fig. 1F, upper lane)^24^. UBXN1 was also localized in cytoplasm, and formed spot-like structures (Fig. 1F, middle lane), which is reportedly attributable to its association with the ubiquitin–proteasome system^27,28^. The coexpression of both proteins caused the accumulation of UBXN1 in the cytoplasm, and its signal localized strongly with that of the V protein (Fig. 1F, lower lane). Furthermore, NiV V was co-localized with endogenous UBXN1 in the cytoplasm (Fig. 1G). These results suggest that V protein interacts intracellularly with UBXN1.

### Identification of the binding domains of NiV V and UBXN1

**Figure 2:**
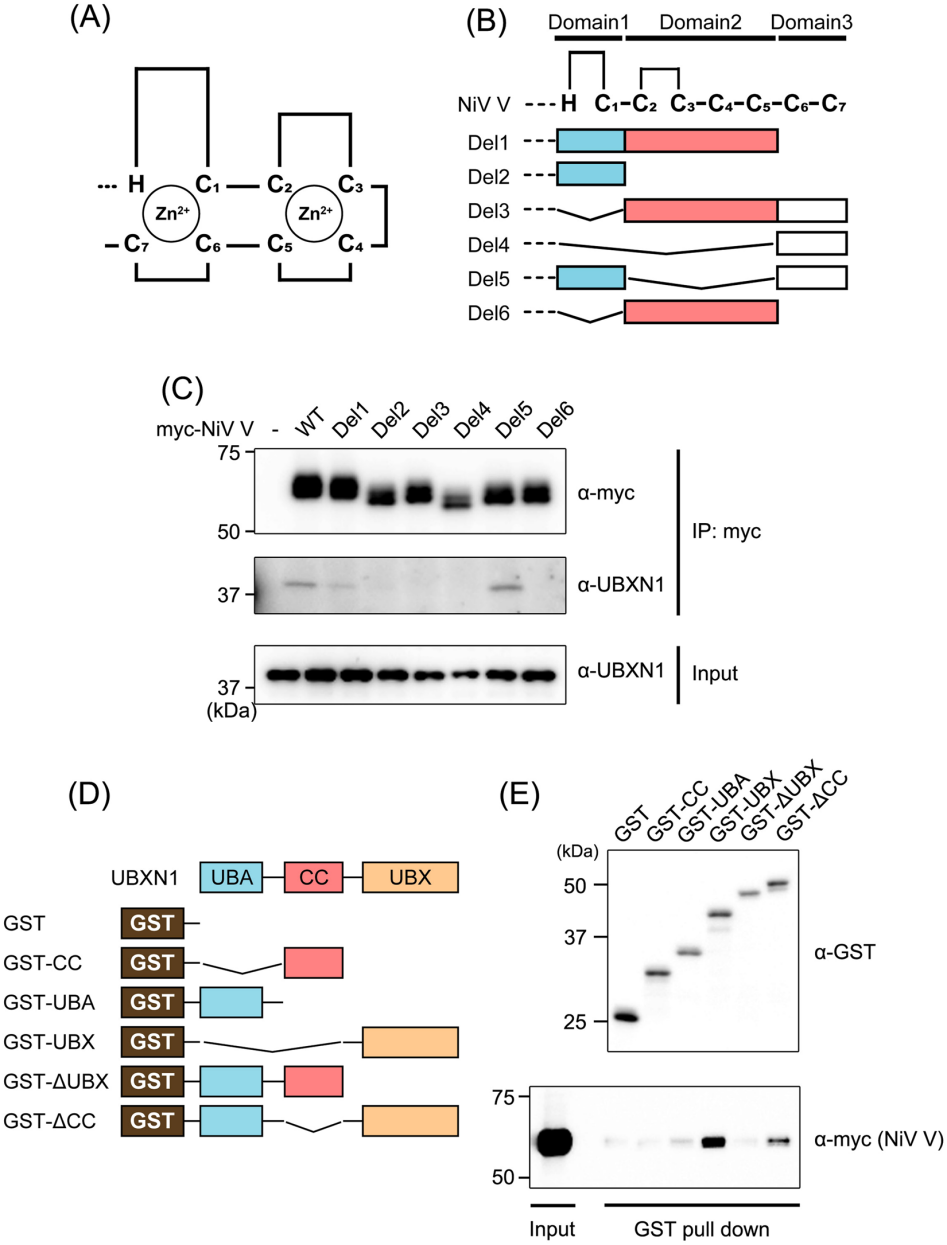
Identification of the binding domains of NiV V and UBXN1. (**A**,**B**) Schematic diagrams of the C-terminal domain of V protein (**A**) and its deletion mutants (**B**) are shown. (**C**) Wild-type NiV V and deletion mutants of NiV V were expressed in HEK293T cells, and an immunoprecipitation assay and western blotting were performed as described in Fig. 1B. (**D**) A schematic diagram of GST-tagged deletion mutants of UBXN1 is shown. (**E**) The interactions between GST-tagged UBXN1 mutants and myc-tagged NiV V were evaluated with a GST pull-down assay. Pulled-down proteins were detected with western blotting. The blots presented in (**C**,**E**) were cropped from different images to improve clarity. Full-length blots are presented in Supplementary Figure S1.

The V protein contains two cysteine-rich zinc-finger motifs in its C-terminal domain (Fig. 2A), which are conserved among the paramyxoviruses^29^. To determine the binding domain of NiV V, the C-terminal domain was divided into three subdomains (Domain1–3) and vectors expressing various deletion mutants lacking each subdomain of NiV V (Del1–6), were generated (Fig. 2B). In an immunoprecipitation assay, the binding affinity of NiV V for UBXN1 was detected with Del1 and Del5, but not with Del2, -3, -4, or -6 (Fig. 2C). The results indicate that Domain1 is critical for the interaction of the two proteins. However, because Del2 did not coimmunoprecipitate with UBXN1, and the binding capacity of Del5 for UBXN1 was as strong as that of wild-type NiV V protein, whereas that of Del1 was much weaker, the intact structure of the proximal zinc-finger motif, including Domain1 and Domain3, must be important for the efficient interaction of the V protein with UBXN1. It has been reported that UBXN1 consists of three characterized domains, a ubiquitin-associated (UBA) domain, a coiled-coil (CC) domain, and a ubiquitin regulatory X (UBX) domain^27,28^. To determine the V-binding domain of UBXN1, expression vectors for GST-tagged deletion mutants of UBXN1 were generated: GST-CC, -UBA, -UBX, -ΔUBX, and -ΔCC, (Fig. 2D). In a GST pull-down assay, the V protein was pulled down by two mutants of UBXN1, GST-UBX and -ΔCC, both of which contained the UBX domain, but not by the other mutants (Fig. 2E). This result suggests that NiV V interacts with the UBX domain of UBXN1.

### NiV V increases the stability of UBXN1

**Figure 3:**
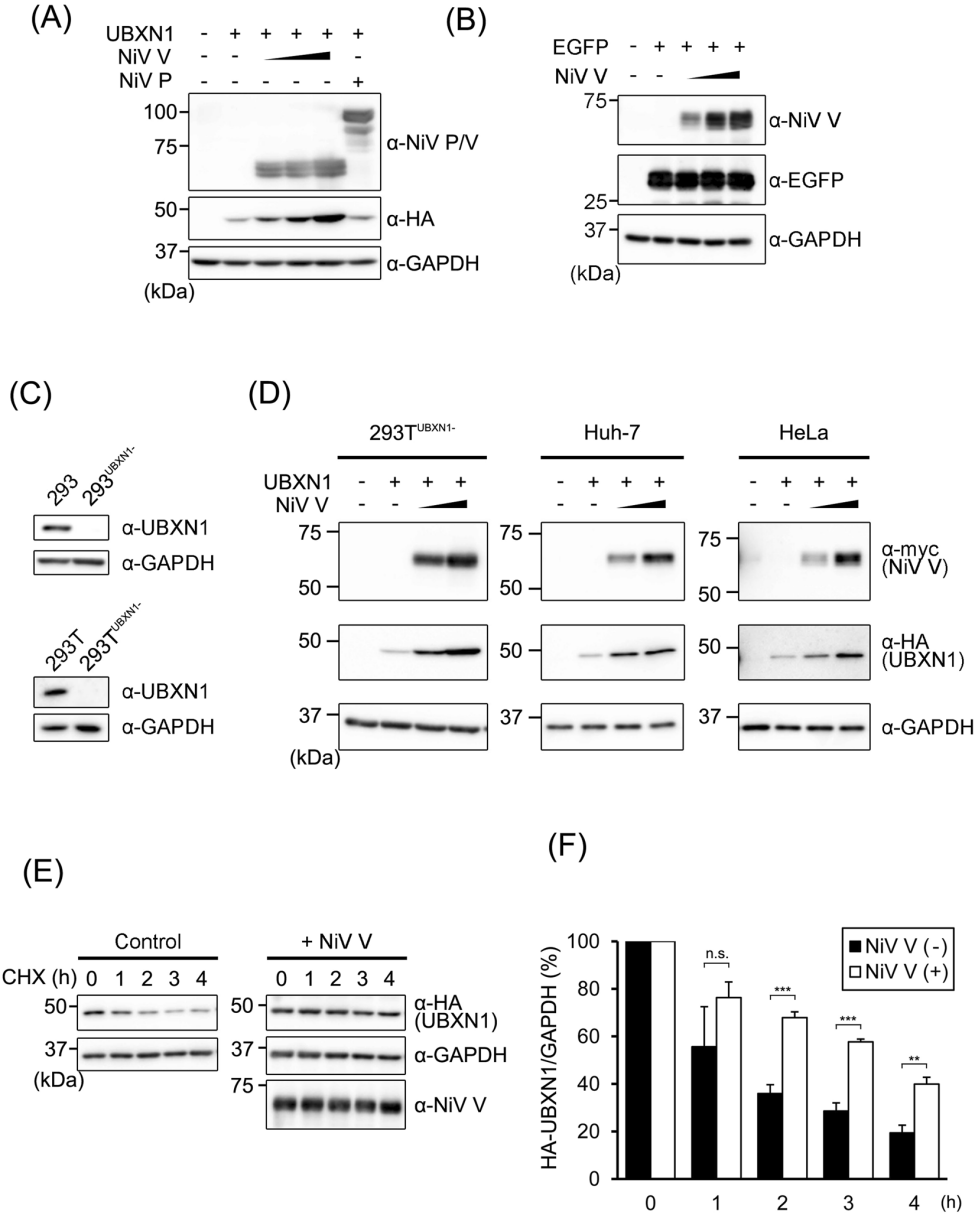
NiV V stabilizes UBXN1. (**A**) HEK293T cells were transfected with 400 ng of a vector expressing HA-tagged UBXN1 together with various amounts (100 ng, 200 ng, or 400 ng) of vector expressing NiV V or 400 ng NiV P. The total amount of transfected vector was kept constant by the addition of empty vector. After 24 h, the cells were lysed and the proteins were detected with western blotting. (**B**) HEK293T cells were transfected with 400 ng of a vector expressing for EGFP together with various amounts of vector expressing NiV V as described in (**A**). The proteins were detected with western blotting. (**C**) UBXN1 genes in HEK293 and 293 T cells were knocked out by the CRISPR-Cas9 system. The depletion of UBXN1 in 293^UBXN1−^ and 293T^UBXN1−^ cells was verified with western blotting. (**D**) 293T^UBXN1−^, Huh-7 and HeLa cells were transfected with 400 ng of a vector expressing HA-tagged UBXN1 together with various amounts (200 ng or 400 ng) of vector expressing NiV V. The total amount of transfected vector was kept constant by the addition of empty vector. After 24 h, the cells were lysed and the proteins were detected with western blotting. (**E**) HEK293T cells were transfected with 400 ng of a vector expressing for HA-tagged UBXN1 together with 400 ng of a vector expressing NiV V or an empty vector. Then the cells were treated with CHX for 0, 1, 2, 3, or 4 h, and the amount of proteins were evaluated with western blotting. (**F**) HA-tagged UBXN1 was expressed with or without NiV V in HEK293T cells. Then, the cells were treated with CHX, and the expression amount of HA-UBXN1 and GAPDH was quantitated as described in (**E**). The CHX assay was repeated three times, and the intensities of the bands were measured and summarized. Error bars indicate standard deviations (N = 3). **P < 0.01, ***P < 0.001, not significant (n.s.) on Student’s *t* test. The blots presented in (**A**–**E**) were cropped from different images to improve clarity. Full-length blots are presented in Supplementary Figure S2.

We transfected the vector expressing HA-tagged UBXN1 together with that expressing NiV V or NiV P. The expression of UBXN1 protein increased, depending on the amount of the NiV V expression vector transfected, but not for NiV P (Fig. 3A). The expression of enhanced green fluorescent protein (EGFP) was not affected by the NiV V expression (Fig. 3B), indicating that NiV V specifically increased the expression of UBXN1. To examine the possibility that a high quantity of UBXN1 results in the increase of its expression amount, we generated the HEK293 cell lines whose UBXN1 gene was knocked-out by a genome editing (Fig. 3C). As previously reported, the induction of IFNβ was increased by the depletion of UBXN1 in both cell lines (Fig. S5A,B). We found that NiV V increased the expression amount of UBXN1 in 293T^UBXN1−^ cells, indicating that the effects of NiV V on the UBXN1 expression is not due to the artifacts derived from a high quantity of UBXN1 (Fig. 3D). Furthermore, this functional effect of NiV V was also observed in various cell types including Huh-7 and HeLa cells (Fig. 3D). Since the mRNA levels of UBXN1 were not affected by NiV V (data not shown), we speculated that NiV V blocks the degradation of UBXN1. Therefore, a cycloheximide (CHX) chase assay was performed to evaluate the rate of UBXN1 proteolysis when the *de novo* protein synthesis was blocked. When NiV V was expressed together with UBXN1, the degradation of UBXN1 was inhibited (Fig. 3E,F). This result suggests that NiV V increases the stability of UBXN1, causing its intracellular accumulation.

### Stabilization of UBXN1 requires the interacting domains of NiV V and UBXN1

**Figure 4:**
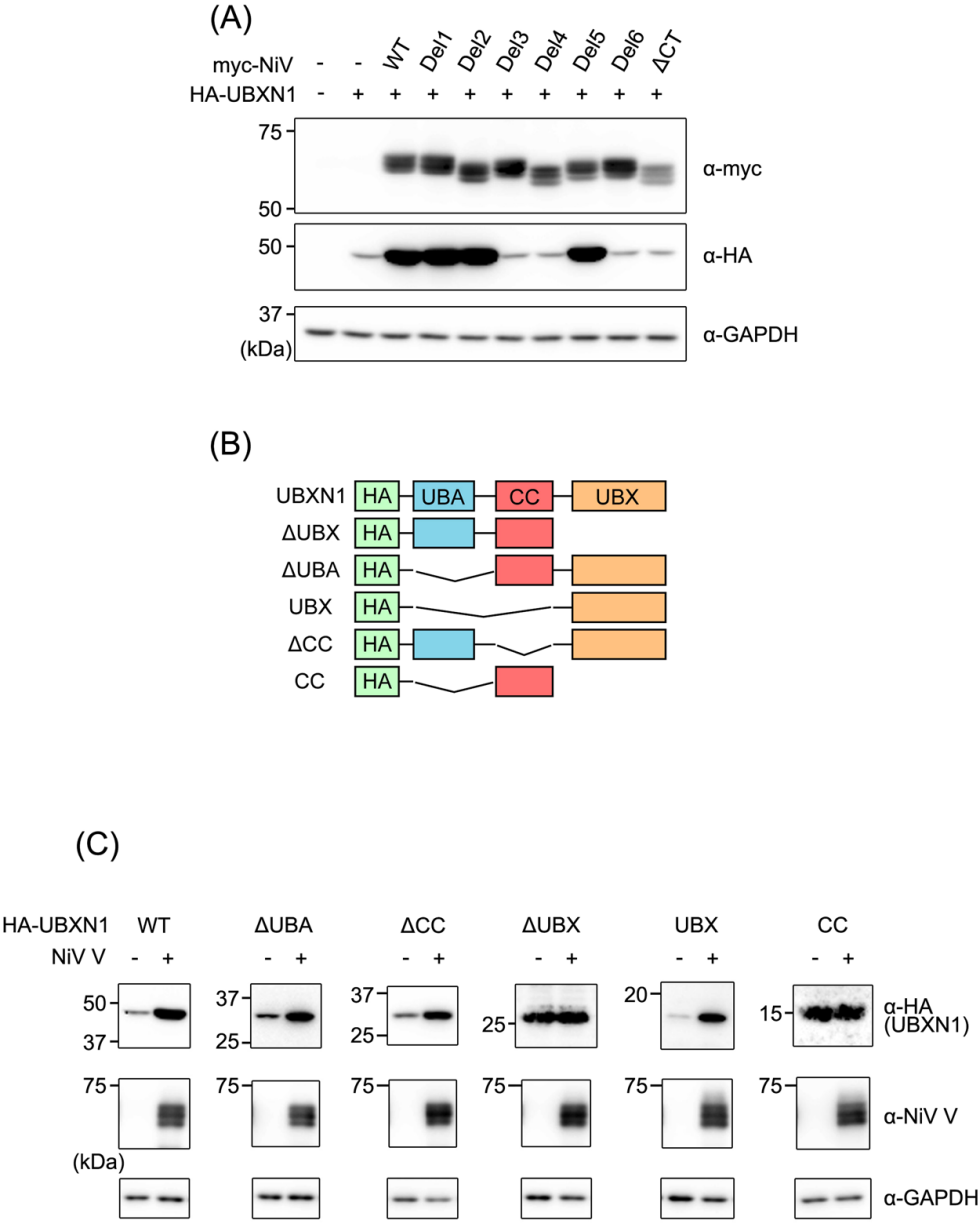
Identification of the domains required to stabilize UBXN1. (**A**) HEK293T cells were transfected with equal amounts of vector expressing HA-tagged UBXN1 and vectors expressing myc-tagged wild-type NiV V or its deletion mutants. At 48 h posttransfection, the proteins were detected with western blotting. (**B**) A schematic diagram of the HA-tagged deletion mutants of UBXN1 is shown. (**C**) HEK293T cells were transfected with vectors expressing HA-tagged deletion mutants of UBXN1 together with a vector expressing NiV V or the empty vector. At 48 h posttransfection, the proteins were detected with western blotting. The blots presented in (**A**,**C**) were cropped from different images to improve clarity. Full-length blots are presented in Supplementary Figure S3.

To determine the domain of V required for the stabilization of UBXN1, the deletion mutants of NiV V described in Fig. 2B was expressed together with UBXN1. The mutants containing Domain1 (Del1, -2 and -5) clearly increased the expression level of UBXN1, whereas the others did not (Fig. 4A). To determine the domain of UBXN1 required for its stabilization by NiV V, we generated various vectors expressing HA-tagged deletion mutants of UBXN1, ΔUBX, ΔUBA, UBX, ΔCC, and CC (Fig. 4B). These mutants were expressed with or without NiV V. The expression levels of the deletion mutants containing the UBX domain (ΔUBA, UBX, and ΔCC) increased when coexpressed with NiV V (Fig. 4C), but the others did not. These results indicate that the stabilization of UBXN1 requires Domain1 of NiV V and the UBX domain of UBXN1, which are identical to the domains required for the interaction of the two proteins (Fig. 2C,E). Therefore, we infer that the stabilization of UBXN1 is caused by its interaction with NiV V.

### Amino acids of NiV V required for its interaction with UBXN1

**Figure 5:**
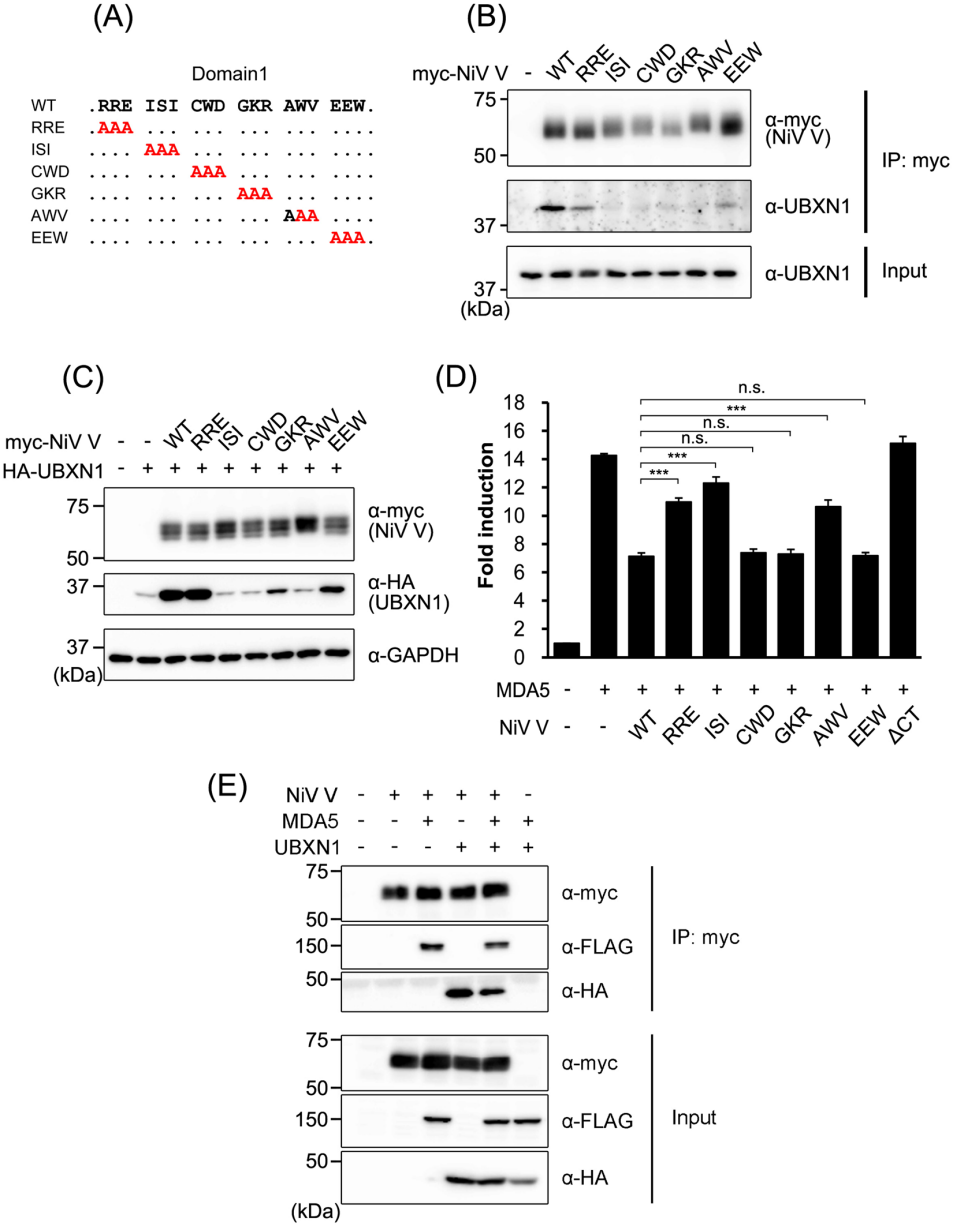
Amino acids in NiV V required for its interaction with UBXN1. (**A**) Schematic diagrams of the alanine-substitution mutants of NiV V are shown. (**B**) Myc-tagged wild-type NiV V and its alanine-substitution mutants were expressed in HEK293T cells, and an immunoprecipitation assay and western blotting were performed as described in Fig. 1B. (**C**) HEK293T cells were transfected with equal amounts of vector expressing HA-tagged UBXN1 and vector expressing myc-tagged wild-type NiV V or its alanine-substitution mutants. At 48 h posttransfection, the proteins were detected with western blotting. (**D**) HEK293T cells were transfected with an IFNβ reporter vector together with vectors expressing FLAG-tagged MDA5 and wild-type NiV V or its alanine-substitution mutants. The total amount of transfected vector was kept constant by the addition of empty vector. At 24 h posttransfection, a luciferase assay was performed. (**E**) HEK293T cells were transfected with vectors expressing myc-tagged NiV V, FLAG-tagged MDA5 and HA-tagged UBXN1. At the 48 h posttransfection, an immunoprecipitation assay was performed with anti-myc antibody. The precipitated proteins were detected with western blotting. Error bars indicate standard deviations (N = 3). ***P < 0.001, not significant (n.s.) on Dunnett’s multiple comparison test. The blots presented in (**B**,**C**,**E**) were cropped from different images to improve clarity. Full-length blots are presented in Supplementary Figure S4.

Ramachandran and Horvath reported that Domain1 of the V protein contains the amino acids required for MDA5 interference^29^. In this study, we found that Domain1 of the V protein also interacts with UBXN1 (Figs 2C and 5A), so we considered whether the same region in Domain1 functions in both MDA5 interference and the interaction with UBXN1. To evaluate the amino-acid requirements for these two functions, we generated vectors expressing various myc-tagged alanine-substitution mutants of NiV V, RRE, ISI, CWD, GKR, AWV, and EEW (Fig. 5A). The mutants were expressed in HEK293T cells and an immunoprecipitation assay was performed. Mutants RRE and EEW maintained the interaction with UBXN1, although it was weaker than that of intact NiV V. However, the interactions of mutants ISI, CWD, GKR, and AWV were eliminated (Fig. 5B). To confirm the stabilization of UBXN1 by the myc-tagged alanine-substitution mutants of NiV V, these mutants were coexpressed with HA-tagged UBXN1. RRE stabilized UBXN1 as effectively as did wild-type NiV V, but GKR and EEW stabilized it less effectively, and ISI, CWD, and AWV did not stabilize it at all (Fig. 5C). Therefore, residues ISI, CWD, and AWV are important for UBXN1 stabilization. To determine the amino acids required for MDA5 interference, the alanine-substitution mutants of NiV V were coexpressed with MDA5 in HEK293T cells, and the induction of IFN was monitored with an IFNβ reporter vector. MDA5 induced the activation of the *IFNB* promoter, and wild-type NiV V interrupted MDA5 activity (Fig. 5D). The alanine-substitution mutants CWD, GKR, and EEW reduced the activity of MDA5 as strongly as did wild-type NiV V, whereas the interference activities of RRE, ISI, and AWV were weaker than that of wild-type NiV V (Fig. 5D). These results suggest that MDA5 and UBXN1 share the amino acid residues in ISI and AWV to interact with NiV V. On the other hand, CWD and GKR contains the amino acid residues specifically critical for the interaction with UBXN1, and RRE contains those for the interaction with MDA5. Since the binding sites to UBXN1 and MDA5 in NiV V were close, we evaluated the competitive binding of these proteins with immunoprecipitation assay. Although the coimmunoprecipitation of UBXN1 and MDA5 was slightly decreased in the competitive binding, both proteins were coimmunoprecipitated with NiV V together (Fig. 5E). These results indicated that the binding sites of NiV V to MDA5 and UBXN1 were close but not identical, and NiV V could interact with both proteins together.

### Stabilized UBXN1 suppresses IFN induction

**Figure 6:**
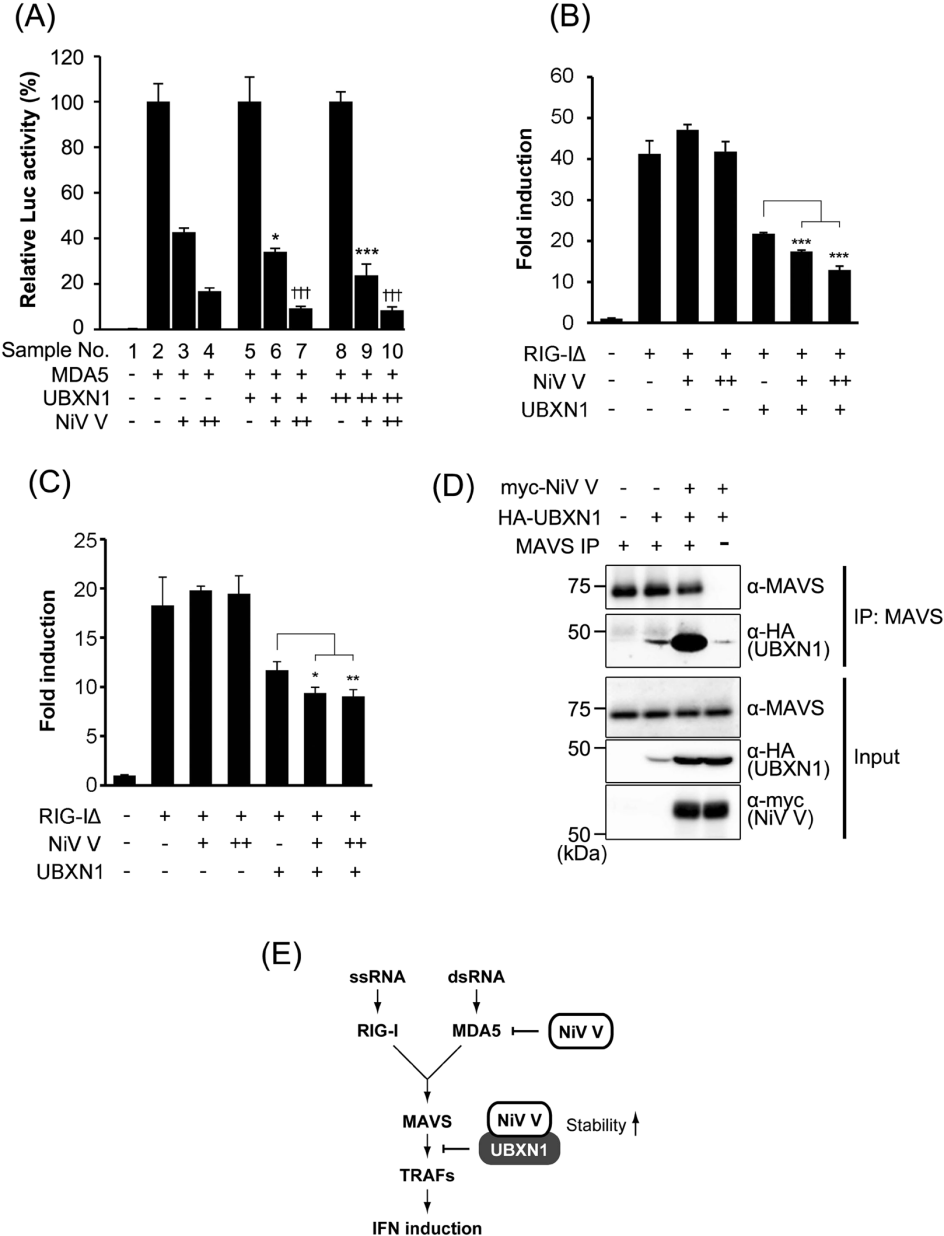
Stabilized UBXN1 suppresses IFN induction. (**A**) HEK293 cells were transfected with an IFNβ reporter vector together with a vector expressing MDA5 and various amounts of vectors expressing NiV V and UBXN1. The total amount of transfected vector was kept constant by the addition of empty vector. At 24 h posttransfection, a luciferase assay was performed. The values in three data sets, sample No. 1–4, 5–7 and 8–10, were normalized by setting the value of sample No. 2, 5 and 8 to 100% respectively. Samples No. 6, 9 and 7, 10 were statistically compared to No. 3 and 4 respectively. The original data without the normalization were shown in Supplementary Figure S5C. (**B**) HEK293 cells were transfected with an IFNβ reporter vector together with vectors expressing RIG-IΔ and NiV V with or without a vector expressing UBXN1. The total amount of transfected vector was kept constant by the addition of empty vector. At 24 h posttransfection, a luciferase assay was performed. (**C**) 293^UBXN1−^ cells were transfected, and the luciferase reporter assay was performed as described in (**B**). (**D**) HA-tagged UBXN1 and myc-tagged NiV V were expressed in HEK293T cells. At 48 h posttransfection, endogenous MAVS was immunoprecipitated with a specific antibody, and the precipitated proteins were detected with western blotting. (**E**) The model of MAVS interference by NiV V suggested by our results is shown. Error bars indicate standard deviations (N = 3). *P < 0.05, **P < 0.01, *** and ^†††^P < 0.001, not significant (n.s.) on Dunnett’s multiple comparison test. The blots presented in (**D**) were cropped from different images to improve clarity. Full-length blots are presented in Supplementary Figure S4.

UBXN1 is reportedly induced in a late step of viral infection, and binds to MAVS, and this binding downregulates the induction of IFN by disrupting the MAVS–TRAF3/6 signaling complex in RIG-I-like receptor signaling^30^. NiV V is known to directly interfere with MDA5, thus suppressing the induction of IFN^18,21^. To examine whether the stabilized UBXN1 increases suppression activity of NiV V on MDA5 signaling, IFN reporter assay was performed. The coexpression of UBXN1 increased MDA5-suppression activity of NiV V, depending on the expression level (Fig. 6A). To examine whether RIG-I signaling was suppressed by the stabilized UBXN1, the constitutively activated RIG-I (RIG-IΔ) was expressed with NiV V and UBXN1, and IFN reporter assay was performed. As previously reported^19^, NiV V did not suppress RIG-I signaling directly (Fig. 6B). However, when coexpressed with UBXN1, NiV V suppressed RIG-I signaling depending on the expression level (Fig. 6B). This suppression effect of NiV V was also observed in 293^UBXN1-^ cells, indicating that it is not due to the artifacts derived from a high amount UBXN1 (Fig. 6C). The immunoprecipitation assay revealed that NiV V increased the interaction between UBXN1 and endogenous MAVS (Fig. 6D). These results suggest that NiV V suppresses the induction of IFN via RIG-I-like receptor signaling by stabilizing UBXN1 (Fig. 6E).

## Discussion

In this study, we newly identified UBXN1 as a protein that interacts with NiV V, and demonstrated that they interact via Domain1 of NiV V and the UBX domain of UBXN1. NiV V increases the stability of UBXN1 by blocking its proteolysis, which requires the interacting domains of both proteins. With alanine-substitution mutants, we showed that Domain1 of NiV V contains the amino acids required both for UBXN1 stabilization and MDA5 interference, but they are not identical. Our results showed that UBXN1 increased the suppression activity of NiV V on MDA5 activation, and conferred the suppression activity on RIG-I activation to NiV V. These results potentially suggest a novel molecular mechanism for the suppression of IFN induction, in which NiV V stabilizes the negative regulator of RIG-I-like receptor signaling, UBXN1.

After recognizing viral RNA in the cytosol, RIG-I and MDA5 recruit MAVS via the CARD–CARD homotypic interaction^31^. The CARD domain of MAVS rapidly forms prion-like aggregates, which interact with downstream signaling proteins, including tumor necrosis factor (TNF) receptor associated factors (TRAFs), resulting in the activation of IKK and TBK1^32^. Wang *et al*. reported that UBXN1 is induced in a late step of viral infection, and negatively regulates RIG-I-like receptor signaling, interacting with the TRAF 3/6-binding site in MAVS through its N-terminal UBA domain as a dominant-negative binder, and thus disrupting MAVS–MAVS aggregation^30^. Our results indicate that NiV V interacts with the C-terminal UBX domain of UBXN1 to stabilize it, suggesting that NiV V potentially enhances the negative regulation of RIG-I-like receptor signaling by stabilizing the induced UBXN1 during viral infection. It has been reported that the V proteins of the paramyxoviruses do not interrupt MAVS directly^22^, but we have shown that NiV V could indirectly interrupts MAVS by stabilizing its dominant-negative binding partner UBXN1. To the best of our knowledge, this is the first report of the interference of MAVS by the V proteins of the paramyxoviruses. It has been reported that V protein targets multiple host proteins to suppress host’s IFN system^18,20–25,33–35^, and therefore we speculate that the ability of V protein to stabilize UBXN1 potentially functions cooperatively with its other abilities such as the direct inhibition to MDA5, LGP2 and STAT proteins to suppress RIG-I- and MDA5-dependent IFN induction and contribute to the pathogenesis.

Our results showed that NiV V increased the amount of UBXN1 expressed by the plasmid transfection, but we could not recognize the increase of endogenous UBXN1 when NiV V was expressed. Our results also indicated that NiV V binds to UBX domain in UBXN1, and it has been reported that multiple host’s proteins such as BRCA1, Homer2 and p97 also bind to UBX domain in UBXN1^27,28,36^. Therefore, we speculate that the only small amount of endogenous UBXN1 in the cells interacted with NiV V because it had already bound to other host proteins, which did not allow us to recognize the functional effects of NiV V on endogenous UBXN1. On the other hand, exogenously expressed UBXN1 might efficiently bind to NiV V before it binds to other host’s proteins, which allow us to clearly show the functional effects of NiV V. It has been reported that the gene expression of UBXN1 is induced by the infection of RNA viruses^30^, which suggests that NiV V potentially interacts with newly synthesized UBXN1 efficiently, and stabilizes it to suppress IFN induction.

It is known that V proteins in paramyxoviruses shares the zinc-finger structure in their C-terminal domains, and interact with common host proteins such as MDA5, LGP2 and STAT proteins^18,19,37–43^. Therefore, paramyxovirus V proteins may also share the affinity to UBXN1. Since the Domain1 in the C-terminal domain of NiV V was identified as a binding site for UBXN1 (Figs 2C and 4A), we evaluated the homology of amino acid sequence in the Domain1 among paramyxoviruses (Supplementary Table 1). The amino acid sequence of HeV V protein was almost identical to that of NiV V (identity: 89%), whereas those of other genera including measles virus (MeV), mumps virus (MuV), Newcastle disease virus (NDV) and Sendai virus (SeV) showed low similarity (identity: 33–50%). It suggests that henipavirus V proteins commonly interact with UBXN1, but other paramyxovirus V proteins might not have a similar interaction with it.

UBXN1 belongs to the family of UBA–UBX-domain-containing proteins (UBXNs). The UBXNs are considered to be the cofactors of an AAA ATPase, p97, which is involved in a large variety of cellular processes, including ubiquitin-dependent proteolysis, the fusion of homotypic membranes, nuclear envelope reassembly, and cell-cycle progression^44^. UBXN1 has been shown to interact with p97 and polyubiquitin, which negatively regulates the proteolysis of ubiquitylated proteins^28,45^. We confirmed the ubiquitylation of NiV V, but the expression levels of NiV V were unaffected by increased amounts of exogenous UBXN1 (data not shown), suggesting that the proteolysis of NiV V is not regulated by UBXN1. The stabilization of UBXN1 by NiV V requires the UBX domain of UBXN1, and the UBX domain adopts the same three-dimensional fold as ubiquitin^46^. Therefore, we speculate that the UBX domain is the determinant of the proteolytic rate of UBXN1, which is recognized by the proteasome system in a similar way to ubiquitin, and that the V protein blocks its recognition, resulting in the stabilization of UBXN1.

It has been reported that mice are resistant to intraperitoneal Henipavirus infection, despite the expression of a functional viral entry receptor. However, the deletion of the type I IFN receptor makes them susceptible to this infection^47^. In cell lines and primary endothelial cells, pretreatment with IFN also inhibited the replication of NiV^14^. These reports suggest the significance of the type I IFN system in controlling lethal Henipavirus infections. A recombinant NiV lacking the V protein induced more IFNβ in primary human microvascular lung endothelial cells in a late step of infection than the wild-type virus^48^, suggesting that the stabilization of UBXN1 by the V protein may contribute to the suppression of IFNβ induction in addition to its many reported IFN-suppressive functions. We believe that the infection experiments using infectious NiV at biosafety level 4 would reveal the role of the potential ability of V protein to stabilize UBXN1 in the viral replication and pathogenesis in future.

## Methods

### Cells

HeLa, Huh-7, HEK293 and HEK293T cells were maintained in Dulbecco’s modified Eagle’s medium (Sigma) supplemented with 10% fetal calf serum under 5% CO2 at 37 °C. Transfection was performed with Lipofectamine LTX (Invitrogen) according to the manufacturer’s instructions. The conditions of transfection for each experiment are not unified.

### Plasmids

To construct the expression vectors for viral proteins, pNiV(6+)^49^ were used as the polymerase chain reaction (PCR) templates. The expression vectors for untagged NiV P and V proteins were constructed by inserting the PCR-amplified cDNA into pcDNA3.1(+) (Invitrogen). The expression vectors for myc-tagged NiV P and V were constructed by inserting the PCR-amplified cDNA into pCMV-myc (Clontech). The deletion mutants of NiV V were constructed with inverted PCR using various combinations of primers, which were phosphorylated with T4 PNK (Toyobo). After electrophoresis and gel extraction, the purified PCR products were self-ligated with T4 DNA ligase (Promega). The alanine-substituted mutants of NiV V were constructed with inverted PCR using various combinations of primers containing the mutated nucleotide sequences. To clone the host genes for use as PCR templates, the total RNA was extracted from HeLa cells with ISOGEN (Nippon Gene), and the cDNA was synthesized by reverse transcription with PrimeScript Reverse Transcriptase (Takara) and an oligo(dT) primer. The expression vector for hemagglutinin (HA)-tagged UBXN1 was constructed by inserting the PCR-amplified cDNA into pCMV-HA (Clontech). The expression vectors for glutathione S-transferase (GST)-tagged UBXN1 were constructed by inserting the PCR-amplified cDNA into pGEX-4T-2 (GE Healthcare). The deletion mutants of GST-tagged UBXN1 were constructed with inverted PCR, as described above. The expression vectors for FLAG-tagged MDA5 and MAVS were constructed by inserting the PCR-amplified cDNA into pFLAG-CMV (Sigma). The expression vector for constitutively active RIG-I (RIG-IΔ) which lacks C-terminal domain corresponding to amino acids 735–925 was constructed by inserting the PCR-amplified cDNA into pcDNA3.1(+). To construct the interferon β (IFNβ) reporter vector, the HeLa genome was extracted with the phenol–chloroform method and used as the PCR template. The promoter region of the IFNB gene, corresponding to nucleotides −300 to +25, was amplified by PCR and inserted into pGL3-Basic (Promega). The expression vector for EGFP (pEGFP-C1) was purchased from Clontech. The vector for the genome editing to deplete UBXN1 gene (px330-UBXN1) was constructed by inserting the cDNA corresponding to 62,678,723–62,678,742 in human chromosome 11 (5′-GAGAAGGCTCTGGCCCTCAC-3′) into the downstream of U6 promoter in px330 vector^50^. PCR was performed with KOD -Plus- Neo DNA polymerase (Toyobo), and the nucleotide sequences of all the constructed vectors were verified with DNA sequencing.

### Immunoprecipitation assay and proteomic analysis

At 48 h posttransfection, the cells were washed twice with phosphate-buffered saline (PBS), and lysed in whole-cell extract buffer (50 mM Tris-HCl [pH 8.0], 280 mM NaCl, 0.5% NP-40, 0.2 mM EDTA, 2 mM EGTA, 10% glycerol, 1 mM dithiothreitol, 1 mM sodium vanadate) supplemented with cOmplete Protease Inhibitor Cocktail (Roche). The cell debris was removed by centrifugation at 17 900 g for 10 min, and the supernatant was collected. For the immunoprecipitation of myc-tagged NiV V and HA-tagged UBXN1, Protein G Sepharose (GE Healthcare) conjugated with equal amounts of anti-myc mouse monoclonal antibody (Clontech), anti-HA mouse monoclonal antibody (Sigma), and mouse control IgG (R&D Systems) was used. For the immunoprecipitation of endogenous MAVS, Protein A Sepharose (GE Healthcare) conjugated with anti-MAVS (CT) antibody (Millipore) was used. For the immunoprecipitation of endogenous UBXN1, Protein A Sepharose (GE Healthcare) conjugated with anti-UBXN1 rabbit polyclonal antibody (Proteintech) was used. After incubation with the cell lysate at 4 °C for 1 h, the beads were washed three times with wash buffer (50 mM Tris-HCl [pH 8.0], 300 mM NaCl, 0.5% NP-40 10% glycerol), and the precipitated proteins were eluted with elution buffer (0.1 M glycine-HCl [pH 2.0]).

The immunoprecipitated proteins were separated with sodium dodecyl sulfate polyacrylamide gel electrophoresis (SDS-PAGE) and detected with silver staining (Apro Science). The bands were excised from the polyacrylamide gel and destained. After digestion with trypsin, the peptides were extracted with 5% (v/v) formic acid and acetonitrile, and analyzed with nanoscale liquid chromatography electrospray tandem mass spectrometry. The detected peptides were analyzed with the Mascot search engine.

### GST pull-down assay

Escherichia coli strain BL21 was transformed with the vector expressing GST-tagged UBXN1, and the expression of the recombinant protein was induced with 1 mM isopropyl β-D-1-thiogalactopyranoside at 30 °C for 3 h. After the bacterial cells were washed three times with PBS, they were lysed and sonicated in lysis buffer (1% Triton X-100, 1% N-lauroylsarcosine in PBS). The cell debris was removed by centrifugation at 15 000 g for 15 min, and the supernatant was incubated with Glutathione Sepharose 4B (GE Healthcare) at 4 °C for 1 h. After the beads were washed three times with wash buffer (described in the “immunoprecipitation assay” section), they were incubated in the cell lysate at 4 °C for 1 h. The beads were washed three times, and the proteins were eluted with elution buffer (25 mM glutathione, 100 mM Tris-HCl [pH 8.9]).

### Western blotting

The proteins in the cell lysate and the eluted product were separated with SDS-PAGE and detected with western blotting. After the proteins were transferred to PVDF membrane, the membrane was cut according to the bands of proteins marker (Precision Plus Protein™ Dual Color Standards, BIO-RAD) to probe multiple proteins. As the primary antibodies, we used anti-myc rabbit polyclonal antibody (Sigma), anti-HA mouse monoclonal antibody (Sigma), anti-HA goat polyclonal antibody (Novus Biologicals), anti-FLAG mouse monoclonal antibody (Sigma), anti-UBXN1 rabbit polyclonal antibody (Millipore), anti-glyceraldehyde 3-phosphate dehydrogenase (GAPDH) mouse monoclonal antibody (Millipore), anti-GST mouse monoclonal antibody (Santa Cruz Biotechnology), anti-MAVS rabbit polyclonal antibody (described in the “immunoprecipitation assay” section), anti-EGFP mouse monoclonal antibody (Clontech), and anti-NiV V rabbit polyclonal antibody, which has been described previously^51^. As the secondary antibodies, horseradish-peroxidase-conjugated goat anti-mouse IgG antibody (Dako), anti-rabbit IgG antibody (Dako), or anti-goat IgG antibody (Dako) were used. Chemiluminescence was detected with ECL Prime Western blotting Detection Reagent (GE Healthcare) and an ImageQuant LAS 4000 biomolecular imager (GE Healthcare).

### Cycloheximide chase

At 24 h posttransfection, the cells were trypsinized and split into five equal portions. After 24 h, the cells were treated with 100 µg/ml cycloheximide (Sigma), and lysed as described in the “immunoprecipitation assay” section.

### Genome editing

The cells were transfected with px330-UBXN1 together with pQCXIP (Clontech), and were treated with 2 µg/ml puromycin (Sigma) for 9 days. After the cells were maintained in the medium without puromycin for 5 days, the depletion of UBXN1 was verified with western blotting analysis.

### Indirect immunofluorescence assay

At 24 h posttransfection, the cells were fixed with 4% paraformaldehyde for 30 min. After the cells were washed three times with PBS, they were incubated in blocking buffer (3% bovine serum albumin, 0.1% Triton X-100 in PBS) at room temperature for 30 min. For the staining of myc-tagged NiV V, UBXN1 and HA-tagged UBXN1, anti-myc rabbit polyclonal antibody (Sigma), anti-myc mouse monoclonal antibody (Clontech), anti-UBXN1 rabbit polyclonal antibody (Millipore) and anti-HA mouse monoclonal antibody (Sigma) were incubated with the cell in blocking buffer at 4 °C for over night. For control staining, rabbit serum was used. After the cells were washed three times with wash buffer (0.05% Tween 20 in PBS), they were incubated with Alexa-Fluor-488-conjugated goat anti-rabbit antibody (Invitrogen), Alexa-Fluor-568-conjugated goat anti-mouse antibody (Invitrogen), and Hoechst 33342 (Cambrex) in blocking buffer at room temperature for 1 h. After the cells were washed three times, their immunofluorescence was observed with an IX70 laser confocal microscope and the FluoView FV1000 system (Olympus).

### Luciferase assay

For the MDA5 and RIG-I signaling assay, the cells were transfected with the IFNβ reporter vector and phRL-TK (Promega) as the internal control, together with vectors expressing MDA5, RIG-IΔ, NiV V and UBXN1. At 24 h posttransfection, the cells were washed once with PBS and lysed in Passive Lysis Buffer (Promega). The luciferase activities in the cell lysates were measured with the Dual Luciferase Assay System (Promega).

### Quantitative PCR assay

The template DNA fragment of the NiV minigenome RNA was described previously^51^. The RNA was synthesized with the T7 RiboMax™ Express Large Scale RNA Production System (Promega) and purified according to the manufacturer’s instructions. After the quality of the synthesized RNA was checked with formaldehyde gel electrophoresis, the RNA was transfected using Lipofectamine 2000 (Invitrogen). Total RNA was extracted from the cells with TRIzol Reagent (Invitrogen), and was reverse-transcribed with PrimeScript Reverse Transcriptase (Takara) and an oligo(dT) primer. The quantitative PCR was performed with THUNDERBIRD® SYBR® qPCR Mix (Toyobo) and specific primers for IFNβ (forward: 5′-CAGGAGAGCAATTTGGAGGA-3′; reverse: 5′-CTTTCGAAGCCTTTGCTCTG-3′) and β-actin (forward: 5′-TGGACTTCGAGCAAGAGATGG-3′; reverse: 5′-GGAAGGAAGGCTGGAAGAGTG-3′).

### Statistical analysis

Quantitative data were presented as means ± standard deviations of triplicate samples. For the assessment of the normality, Kolmogorov-Smirnov test was used. The statistical significance between two groups were examined by Student’s *t* test after their homoscedasticity was assessed by F test. For the multiple comparison, one-way analysis of variance (ANOVA) followed by Dunnett’s multiple comparison test was used. *P* value of <0.05 was considered statistically significant.

## Electronic supplementary material

Supplementary information

## References

[1] Chua KB (2000). Nipah virus: a recently emergent deadly paramyxovirus. Science.

[2] Eaton BT, Broder CC, Middleton D, Wang LF (2006). Hendra and Nipah viruses: different and dangerous. Nat Rev Microbiol.

[3] Arankalle VA (2011). Genomic characterization of Nipah virus, West Bengal, India. Emerg Infect Dis.

[4] Chadha MS (2006). Nipah virus-associated encephalitis outbreak, Siliguri, India. Emerg Infect Dis.

[5] Ching PK (2015). Outbreak of henipavirus infection, Philippines, 2014. Emerg Infect Dis.

[6] Hossain MJ (2008). Clinical presentation of nipah virus infection in Bangladesh. Clin Infect Dis.

[7] Hsu VP (2004). Nipah virus encephalitis reemergence, Bangladesh. Emerg Infect Dis.

[8] Pernet O (2014). Evidence for henipavirus spillover into human populations in Africa. Nat Commun.

[9] Hayman DT (2008). Evidence of henipavirus infection in West African fruit bats. PLoS One.

[10] Iehle C (2007). Henipavirus and Tioman virus antibodies in pteropodid bats, Madagascar. Emerg Infect Dis.

[11] Li Y (2008). Antibodies to Nipah or Nipah-like viruses in bats, China. Emerg Infect Dis.

[12] Harcourt BH (2000). Molecular characterization of Nipah virus, a newly emergent paramyxovirus. Virology.

[13] Yoneda M (2010). The nonstructural proteins of Nipah virus play a key role in pathogenicity in experimentally infected animals. PLoS One.

[14] Satterfield BA (2015). The immunomodulating V and W proteins of Nipah virus determine disease course. Nat Commun.

[15] Satterfield BA (2016). Nipah Virus C and W Proteins Contribute to Respiratory Disease in Ferrets. J Virol.

[16] Mathieu C (2012). Nonstructural Nipah virus C protein regulates both the early host proinflammatory response and viral virulence. J Virol.

[17] Marsh GA (2012). Cedar virus: a novel Henipavirus isolated from Australian bats. PLoS Pathog.

[18] Andrejeva J (2004). The V proteins of paramyxoviruses bind the IFN-inducible RNA helicase, mda-5, and inhibit its activation of the IFN-beta promoter. Proc Natl Acad Sci USA.

[19] Childs K (2007). mda-5, but not RIG-I, is a common target for paramyxovirus V proteins. Virology.

[20] Davis ME (2014). Antagonism of the phosphatase PP1 by the measles virus V protein is required for innate immune escape of MDA5. Cell Host Microbe.

[21] Childs K, Randall R, Goodbourn S (2012). Paramyxovirus V proteins interact with the RNA Helicase LGP2 to inhibit RIG-I-dependent interferon induction. J Virol.

[22] Kitagawa Y (2011). A tryptophan-rich motif in the human parainfluenza virus type 2 V protein is critical for the blockade of toll-like receptor 7 (TLR7)- and TLR9-dependent signaling. J Virol.

[23] Shaw ML, Cardenas WB, Zamarin D, Palese P, Basler CF (2005). Nuclear localization of the Nipah virus W protein allows for inhibition of both virus- and toll-like receptor 3-triggered signaling pathways. J Virol.

[24] Rodriguez JJ, Parisien JP, Horvath CM (2002). Nipah virus V protein evades alpha and gamma interferons by preventing STAT1 and STAT2 activation and nuclear accumulation. J Virol.

[25] Rodriguez JJ, Wang LF, Horvath CM (2003). Hendra virus V protein inhibits interferon signaling by preventing STAT1 and STAT2 nuclear accumulation. J Virol.

[26] Sleeman K (2008). The C, V and W proteins of Nipah virus inhibit minigenome replication. J Gen Virol.

[27] McNeill H, Knebel A, Arthur JS, Cuenda A, Cohen P (2004). A novel UBA and UBX domain protein that binds polyubiquitin and VCP and is a substrate for SAPKs. Biochem J.

[28] Ishibashi T (2005). A novel protein specifically interacting with Homer2 regulates ubiquitin-proteasome systems. J Biochem.

[29] Ramachandran A, Horvath CM (2010). Dissociation of paramyxovirus interferon evasion activities: universal and virus-specific requirements for conserved V protein amino acids in MDA5 interference. J Virol.

[30] Wang P (2013). UBXN1 interferes with Rig-I-like receptor-mediated antiviral immune response by targeting MAVS. Cell Rep.

[31] Bowie AG, Unterholzner L (2008). Viral evasion and subversion of pattern-recognition receptor signalling. Nat Rev Immunol.

[32] Hou F (2011). MAVS forms functional prion-like aggregates to activate and propagate antiviral innate immune response. Cell.

[33] Shaw ML, Garcia-Sastre A, Palese P, Basler CF (2004). Nipah virus V and W proteins have a common STAT1-binding domain yet inhibit STAT1 activation from the cytoplasmic and nuclear compartments, respectively. J Virol.

[34] Rodriguez KR, Horvath CM (2014). Paramyxovirus V protein interaction with the antiviral sensor LGP2 disrupts MDA5 signaling enhancement but is not relevant to LGP2-mediated RLR signaling inhibition. J Virol.

[35] Irie T, Kiyotani K, Igarashi T, Yoshida A, Sakaguchi T (2012). Inhibition of interferon regulatory factor 3 activation by paramyxovirus V protein. J Virol.

[36] Wu-Baer F, Ludwig T, Baer R (2010). The UBXN1 protein associates with autoubiquitinated forms of the BRCA1 tumor suppressor and inhibits its enzymatic function. Mol Cell Biol.

[37] Kubota T, Yokosawa N, Yokota S, Fujii N (2001). C terminal CYS-RICH region of mumps virus structural V protein correlates with block of interferon alpha and gamma signal transduction pathway through decrease of STAT 1-alpha. Biochem Biophys Res Commun.

[38] Palosaari H, Parisien JP, Rodriguez JJ, Ulane CM, Horvath CM (2003). STAT protein interference and suppression of cytokine signal transduction by measles virus V protein. J Virol.

[39] Parisien JP (2001). The V protein of human parainfluenza virus 2 antagonizes type I interferon responses by destabilizing signal transducer and activator of transcription 2. Virology.

[40] Parisien JP, Lau JF, Rodriguez JJ, Ulane CM, Horvath CM (2002). Selective STAT protein degradation induced by paramyxoviruses requires both STAT1 and STAT2 but is independent of alpha/beta interferon signal transduction. J Virol.

[41] Takeuchi K, Kadota SI, Takeda M, Miyajima N, Nagata K (2003). Measles virus V protein blocks interferon (IFN)-alpha/beta but not IFN-gamma signaling by inhibiting STAT1 and STAT2 phosphorylation. FEBS Lett.

[42] Ulane CM, Horvath CM (2002). Paramyxoviruses SV5 and HPIV2 assemble STAT protein ubiquitin ligase complexes from cellular components. Virology.

[43] Yokosawa N, Kubota T, Fujii N (1998). Poor induction of interferon-induced 2′,5′-oligoadenylate synthetase (2–5 AS) in cells persistently infected with mumps virus is caused by decrease of STAT-1 alpha. Arch Virol.

[44] Schuberth C, Buchberger A (2008). UBX domain proteins: major regulators of the AAA ATPase Cdc48/p97. Cell Mol Life Sci.

[45] LaLonde DP, Bretscher A (2011). The UBX protein SAKS1 negatively regulates endoplasmic reticulum-associated degradation and p97-dependent degradation. J Biol Chem.

[46] Buchberger A, Howard MJ, Proctor M, Bycroft M (2001). The UBX domain: a widespread ubiquitin-like module. J Mol Biol.

[47] Dhondt KP (2013). Type I interferon signaling protects mice from lethal henipavirus infection. J Infect Dis.

[48] Lo MK (2012). Distinct and overlapping roles of Nipah virus P gene products in modulating the human endothelial cell antiviral response. PLoS One.

[49] Yoneda M (2006). Establishment of a Nipah virus rescue system. Proc Natl Acad Sci USA.

[50] Cong L (2013). Multiplex genome engineering using CRISPR/Cas systems. Science.

[51] Omi-Furutani M, Yoneda M, Fujita K, Ikeda F, Kai C (2010). Novel phosphoprotein-interacting region in Nipah virus nucleocapsid protein and its involvement in viral replication. J Virol.

