# Supplementary material for: Possible role of the Nipah virus V protein in the regulation of the interferon beta induction by interacting with UBX domain-containing protein1
Source: Sci Rep. 2018 May 16;8:7682. doi: 10.1038/s41598-018-25815-9 (PMC5955904; doi:10.1038/s41598-018-25815-9)

Title: Possible role of the Nipah virus V protein in the regulation of the interferon beta induction by interacting with UBX domain-containing protein 1

Authors

Shotaro Uchida, Ryo Horie, Hiroki Sato, Chieko Kai, and Misako Yoneda*

Affiliation

Laboratory Animal Research Center and International Research Center for Infectious Diseases, Institute of Medical Science, The University of Tokyo, Tokyo, Japan

^*^Corresponding author

Mailing address: 4-6-1 Shirokanedai, Minato-ku, Tokyo 108-8639, Japan. Phone: 81-3-5449-5498; Fax: 81-3-5449-5379, E-mail: [yone@ims.u-tokyo.ac.jp](mailto:yone@ims.u-tokyo.ac.jp)

Figure legends

**Supplementary Figure 1. Full-length blots corresponding to Fig. 1-2.** (A-G) The full-length blots of Fig. 1A (A), Fig. 1B (B), Fig. 1C (C), Fig. 1D (D), Fig. 1E (E), Fig. 2C (F) and Fig. 2E (G) were shown.

**Supplementary Figure 2. Full-length blots corresponding to Fig. 3.** (A-E) The full-length blots of Fig. 3A (A), Fig. 3B (B), Fig. 3C (C), Fig. 3D (D) and Fig. 3E (E) were shown.

**Supplementary Figure 3. Full-length blots corresponding to Fig. 4.** (A, B) The full-length blots of Fig. 4A (A) and Fig. 4C (B) were shown.

**Supplementary Figure 4. Full-length blots corresponding to Fig. 5-6.** (A-D) The full-length blots of Fig. 5B (A), Fig. 5C (B), Fig. 5E (C) and Fig. 6D (D) were shown.

**Supplementary Figure 5.** (A, B) 293 and 293^UBXN1-^ cells (A), and 293T and 293T^UBXN1-^ cells (B) were transfected with the NiV minigenome RNA, and the mRNA expression of IFNβ and β-actin was evaluated by a quantitative PCR assay. The expression amount of IFNβ was normalized with that of β-actin. (C) The original luciferase activities in Fig. 6A before the normalization were shown. Error bars indicate standard deviations (N = 3). **P < 0.01, ***P < 0.001 on Student’s *t* test.

**Supplementary Table 1. Amino acid sequence alignment of the binding domain for UBXN1 in V proteins of paramyxoviruses.** The amino acid alignment of Domain1 in various paramyxovirus V proteins was shown. The amino acid identity was calculated by comparing to NiV V. The different amino acid residues from those of NiV V were shown in white letters on a black background.


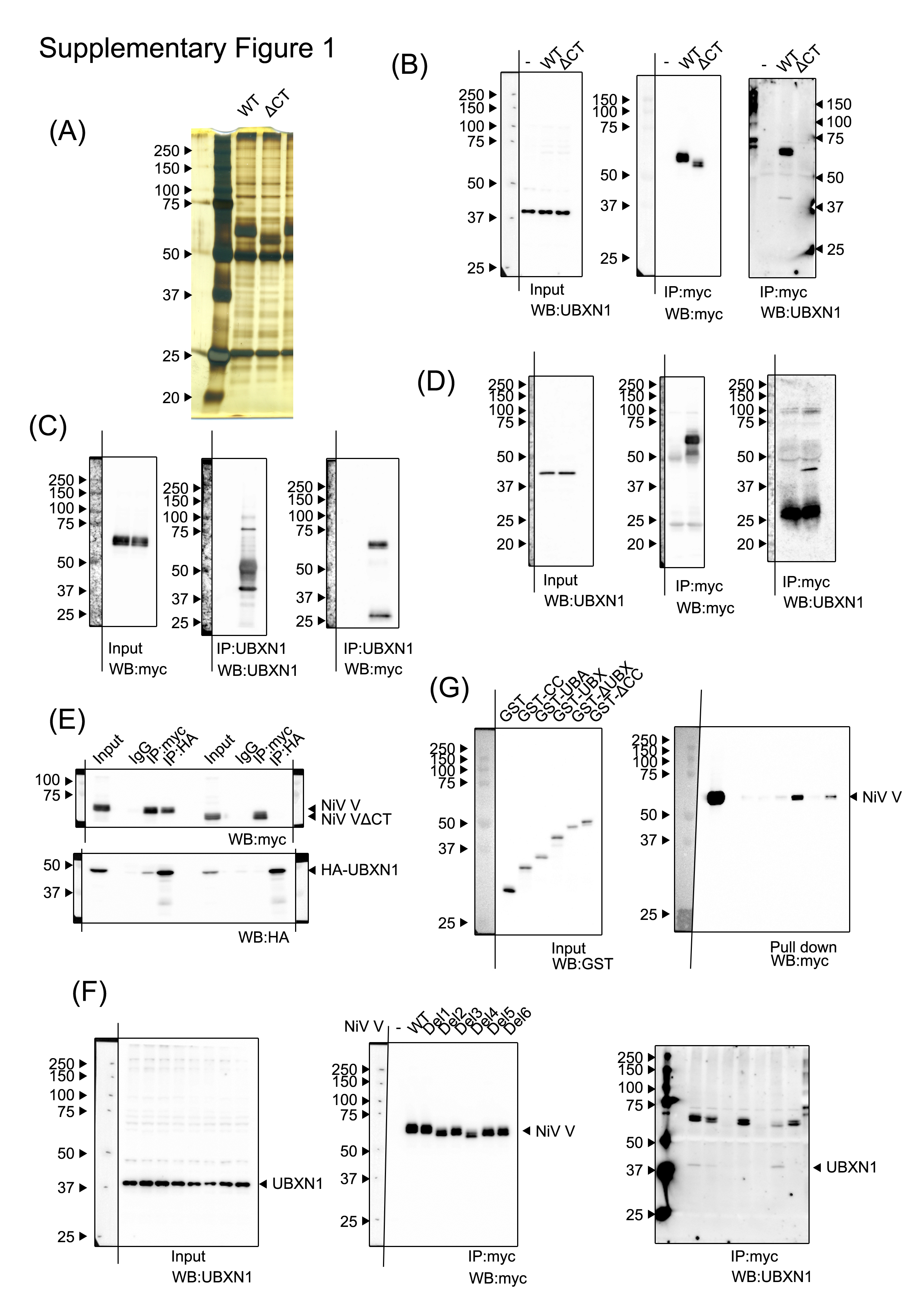


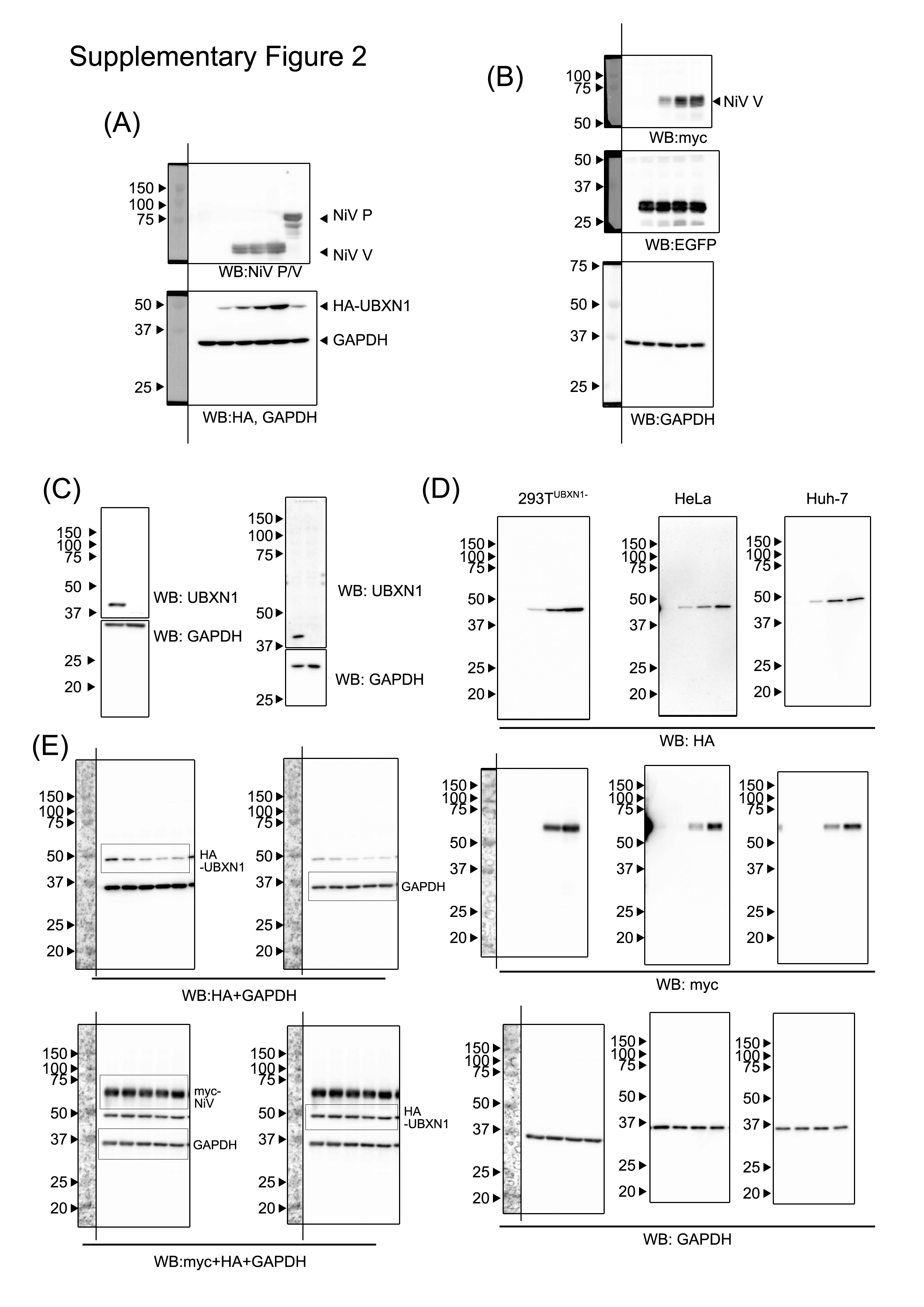


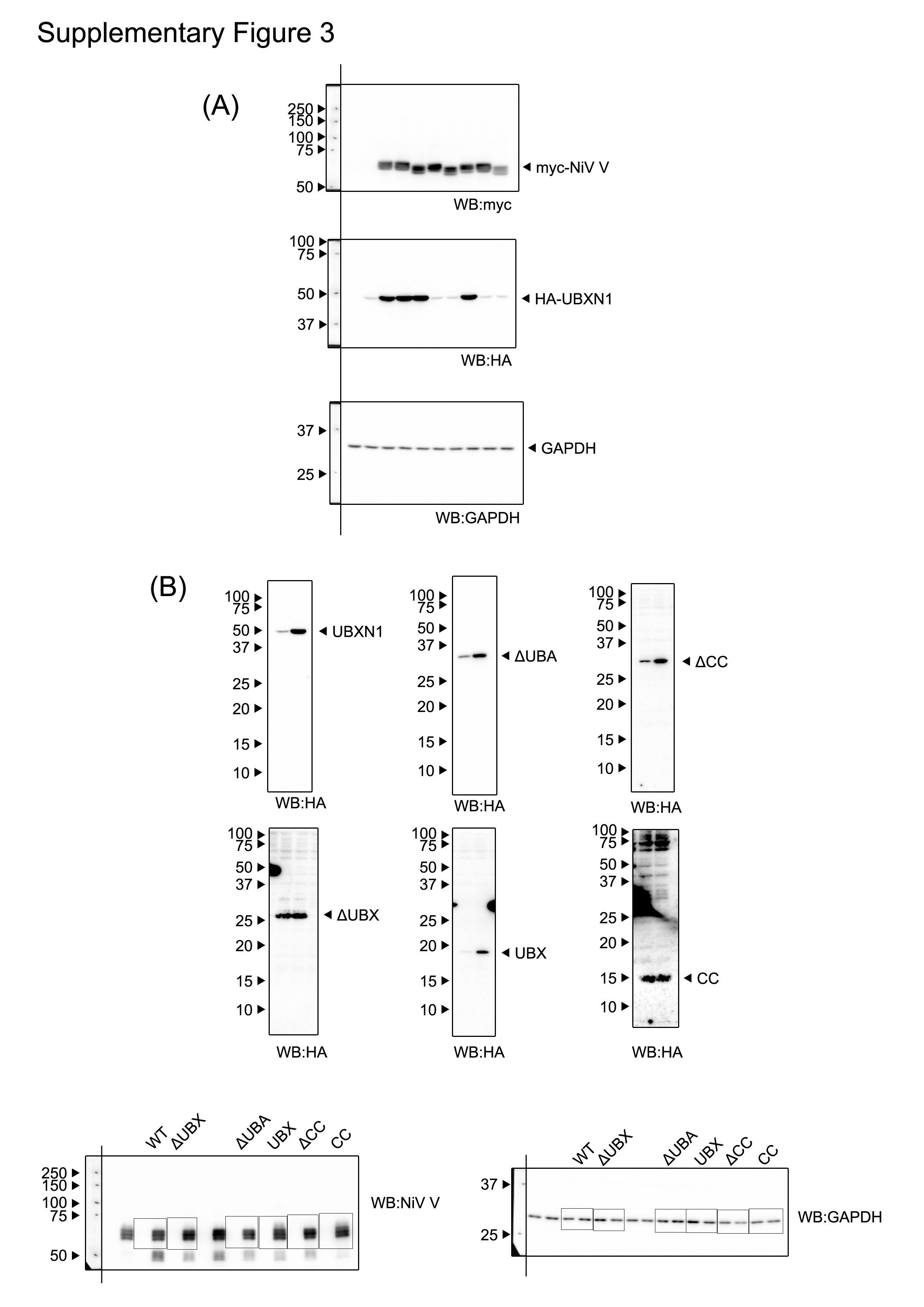


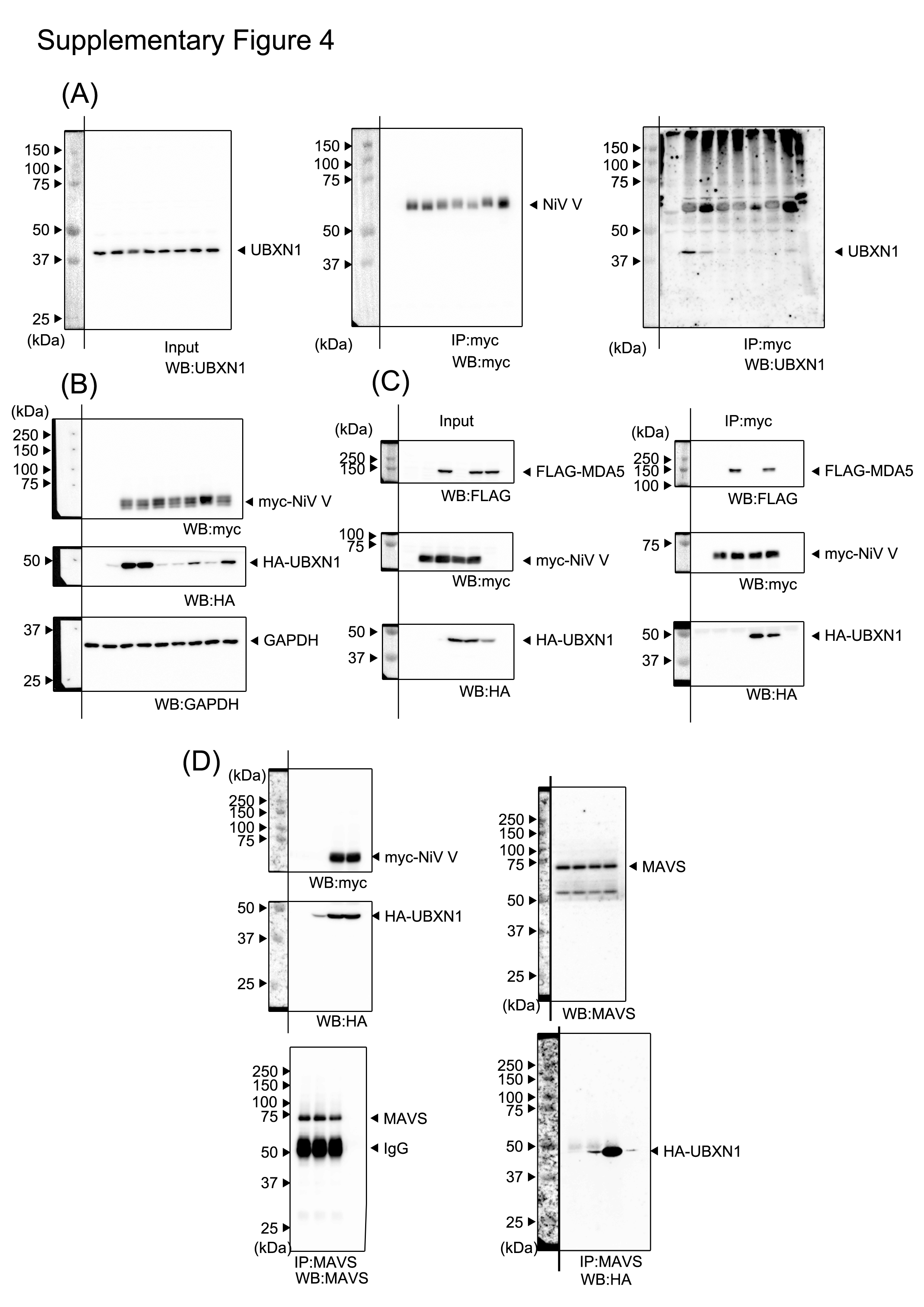


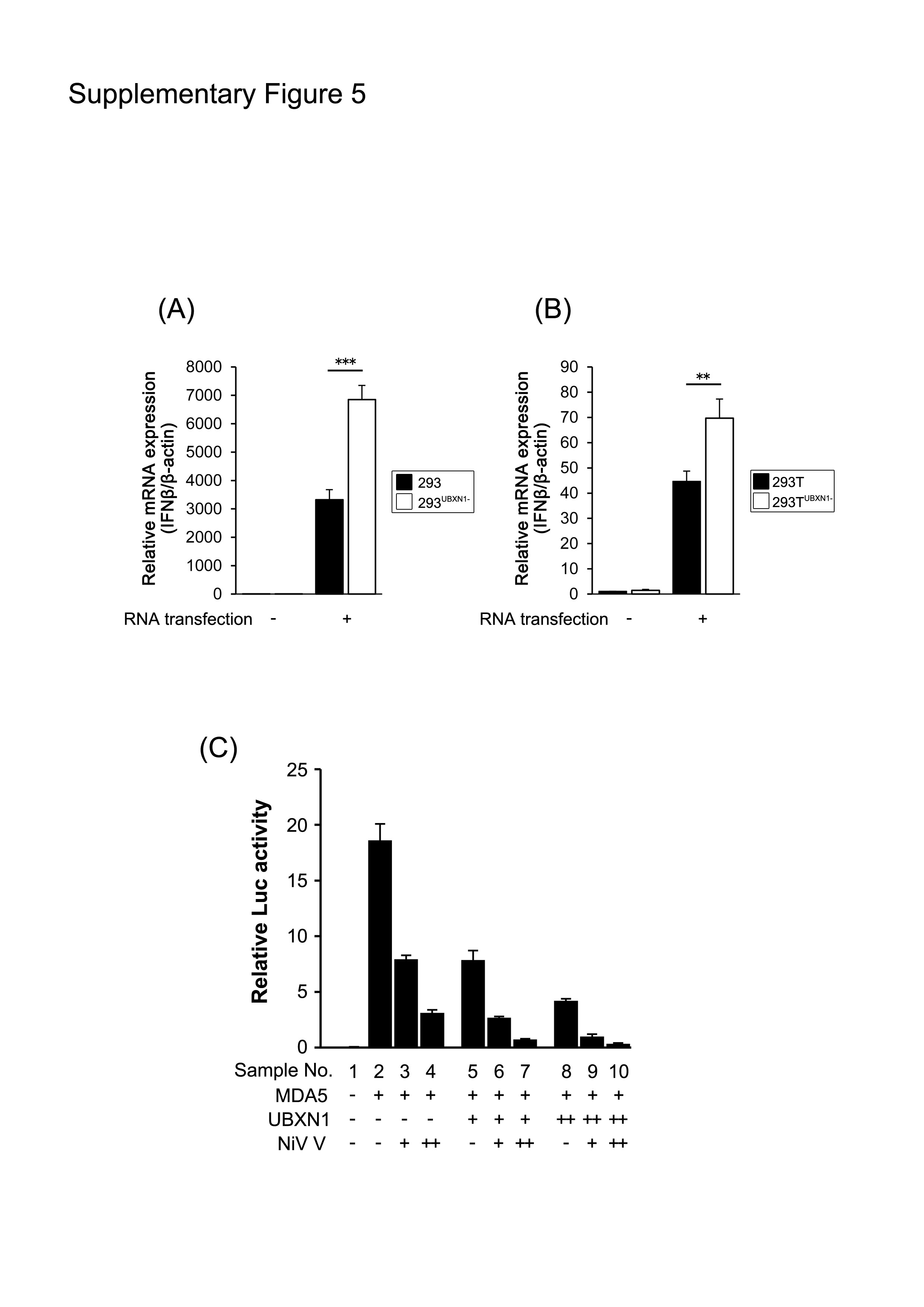


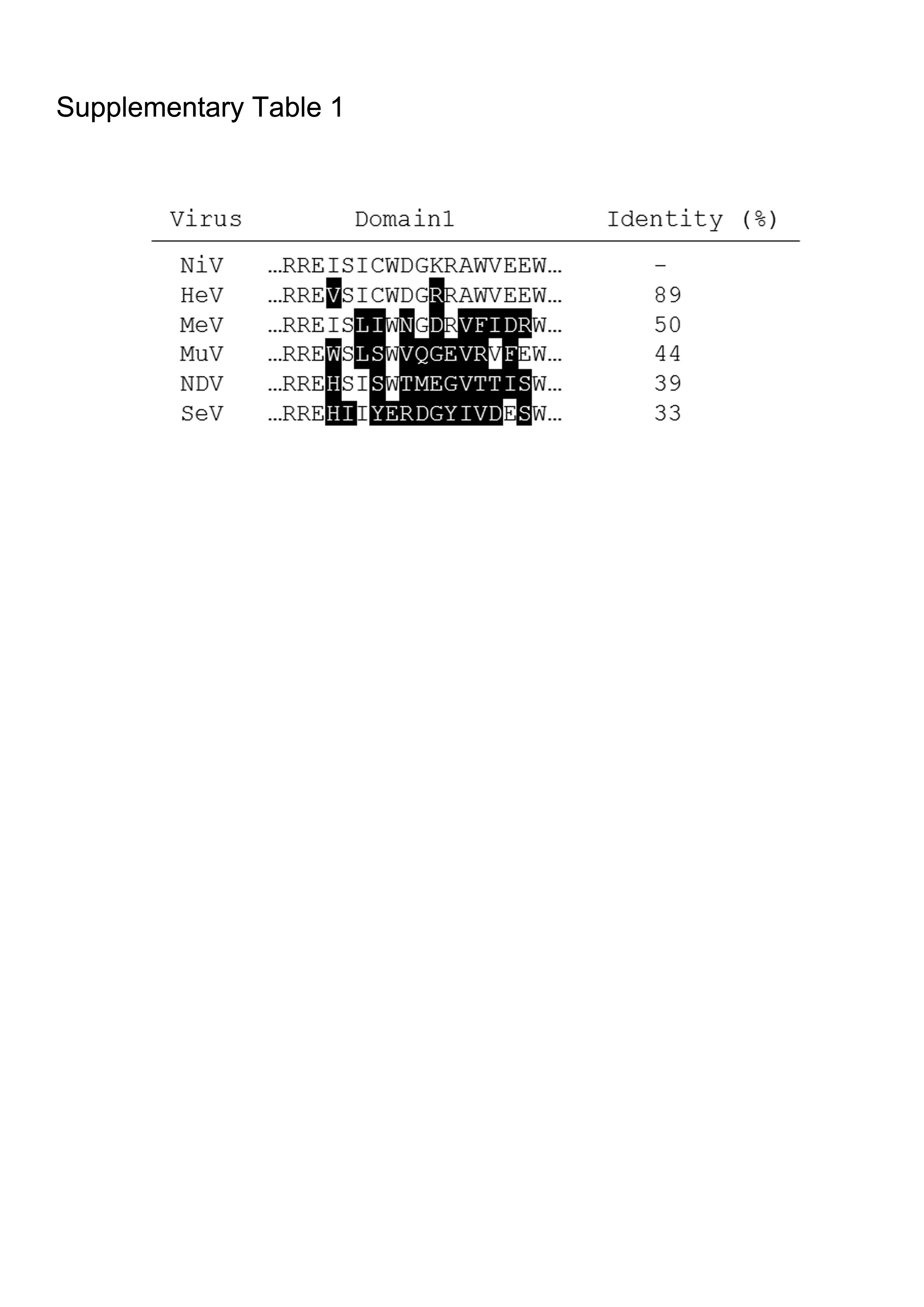

Supplement: Supplementary file 1 — Supplementary information [file 41598_2018_25815_MOESM1_ESM.doc]
